# Functionalized graphene grids with various charges for single-particle cryo-EM

**DOI:** 10.1038/s41467-022-34579-w

**Published:** 2022-11-07

**Authors:** Ye Lu, Nan Liu, Yongbo Liu, Liming Zheng, Junhao Yang, Jia Wang, Xia Jia, Qinru Zi, Hailin Peng, Yu Rao, Hong-Wei Wang

**Affiliations:** 1 School of Life Sciences, Tsinghua University, Beijing, China; 2 Tsinghua-Peking Joint Center for Life Sciences, Tsinghua University, Beijing, China; 3 Beijing Frontier Research Center for Biological Structures, Beijing Advanced Innovation Center for Structural Biology, Tsinghua University, Beijing, China; 4 Ministry of Education Key Laboratory of Protein Sciences, Tsinghua University, Beijing, China; 5 Ministry of Education Key Laboratory of Bioorganic Phosphorus Chemistry & Chemical Biology, Tsinghua University, Beijing, China; 6 School of Pharmaceutical Sciences, Tsinghua University, Beijing, China; 7 Center for Nanochemistry, Beijing Science and Engineering Center for Nanocarbons, Beijing National Laboratory for Molecular Sciences, College of Chemistry and Molecular Engineering, Peking University, Beijing, China; 8 Beijing Graphene Institute, Beijing, China; 9 College of Life Sciences, Wuhan University, Wuhan, Hubei China

**Keywords:** Structure determination, Electron microscopy, Cryoelectron microscopy

## Abstract

A major hurdle for single particle cryo-EM in structural determination lies in the specimen preparation impaired by the air-water interface (AWI) and preferential particle-orientation problems. In this work, we develop functionalized graphene grids with various charges via a dediazoniation reaction for cryo-EM specimen preparation. The graphene grids are paraffin-assistant fabricated, which appear with less contaminations compared with those produced by polymer transfer method. By applying onto three different types of macromolecules, we demonstrate that the high-yield charged graphene grids bring macromolecules away from the AWI and enable adjustable particle-orientation distribution for more robust single particle cryo-EM structural determination.

## Abstract (web summary)

Air-water interface and preferential orientation problems are crucial challenges in cryo-EM specimen preparation. Here, the authors utilize graphene-coated EM grids functionalized by salts with various electrostatic properties, successfully overcoming preferred orientation.

## Introduction

Cryo-electron microscopy (cryo-EM) technique has been successfully applied to reveal the molecular basis of many essential macromolecules at atomic resolution^1^. However, high-quality cryo-specimen preparation still faces many challenges, exemplified by the preferential orientation and air-water interface (AWI) problems^2^, reducing the success rate and efficiency of high-resolution structure determination by cryo-EM. To alleviate the preferential orientation problem, tilting the cryo-specimen during data collection has been applied^3^, which, however, may result in large beam-induced motion and inaccurate defocus estimation during data processing, thereby impairing the high-resolution reconstruction. Many efforts have been also made to solve the preferential orientation as well as AWI problem by introducing supporting films^4–9^, among which graphene membrane is the most promising one.

Graphene^10^, an atomically thin film comprised of sp^2^-bonded carbon atoms, has a superior electrical and thermal conductivity, mechanical strength and negligible background noise. Graphene membrane of sole nature, however, might induce preferential orientation problem of particles adsorbed to its surface^11^. The fabrication of clean graphene grids also remains a practical challenge for cryo-EM specimen preparation. Although the graphene functionalization has been used to specifically capture and anchor target particles to avoid the AWI^6,12–14^, the current protocols of the bioactive functionalization process of graphene, based on the electrophilic reaction or conjugation interaction with the π-π bonds, are sub-optimal due to contamination built-up on the graphene surface during the transfer process or storage.

In this work, to overcome the graphene functionalization and transfer challenges and solve the preferential orientation problem, we develop a method to produce robust graphene grids functionalized with different electrostatic charges, which provide various electrostatic interaction interface for target macromolecules to bind in and enrich orientations. Applied onto 20S proteasome and ribosome complex, the modified graphene with different charge properties enables particle adsorption on the surface with favorite orientation distribution, thus solving the AWI and preferred orientation problems in cryo-EM specimen. Finally, we successfully determine the cryo-EM structure of Ll.LtrB RNP at 3.2-Å resolution, the highest resolution of the endogenously purified group II intron RNP, whose structural reconstruction is hindered by severe preferential orientation problem on conventional EM grids.

## Results

### Paraffin-aided transfer method enabled clean graphene grid fabrication

As the clean transfer of graphene remains one of the major practical barriers for the application of graphene-based supporting films in cryo-EM, we aimed to develop a robust procedure to transfer graphene on EM grids without contamination. We found that paraffin is an ideal transfer mediator. Paraffin is a chemically inert alkane in solid state with relatively lower melting temperature (50–60 °C) compared to other commonly used transfer mediator such as PMMA, which is normally dissolved and removed by organic solvents. Moreover, the graphene-adsorption energy of paraffin is reported to be much smaller than PMMA^15^, theoretically making it easier to be cleaned from the graphene surface.

**Fig. 1:**
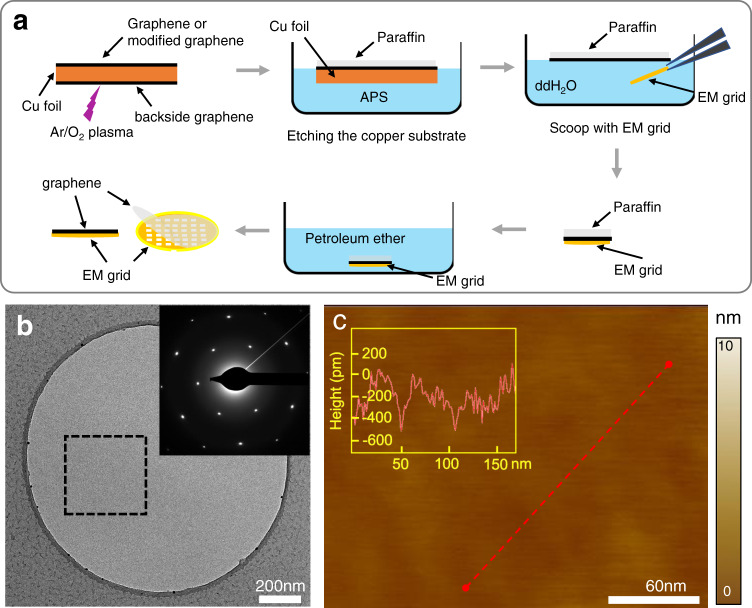
Paraffin-assisted graphene transfer on EM grids. **a** Scheme of paraffin-assisted graphene transfer onto EM grids. **b** A high-magnification TEM image of graphene grid transferred by paraffin. The inset was the selected area electron diffraction (SAED) pattern of the boxed region. Similar results have been repeated in 4 grids. **c** A high-magnification AFM image. The inset was the height profile of the suspended graphene along the red dotted line. Source data are provided as a Source Data file.

In our setup, we first synthesized single-layer graphene on copper foil by CVD method^16^ and then used paraffin to support the graphene during the fabrication procedure (Fig. 1a). The paraffin was then removed by petroleum ether from the graphene surface. In such a setup, we normally can prepare tens of graphene-coated EM grids at once (Supplementary Fig. 1). The sharp diffraction spots under TEM indicating the presence of single-crystal graphene, with significantly reduced contaminations compared with PMMA-assistant graphene transfer method, where 87% holes displayed no or no-more-than-five contamination spots (Fig. 1b and Supplementary Fig. 2), suitable for high-quality cryo-EM specimen preparation. The graphene surface was fairly flat with the roughness of less than 1 nm under AFM (Fig. 1c and Supplementary Fig. 3).

### Preparation and characterization of functionalized graphene

**Fig. 2:**
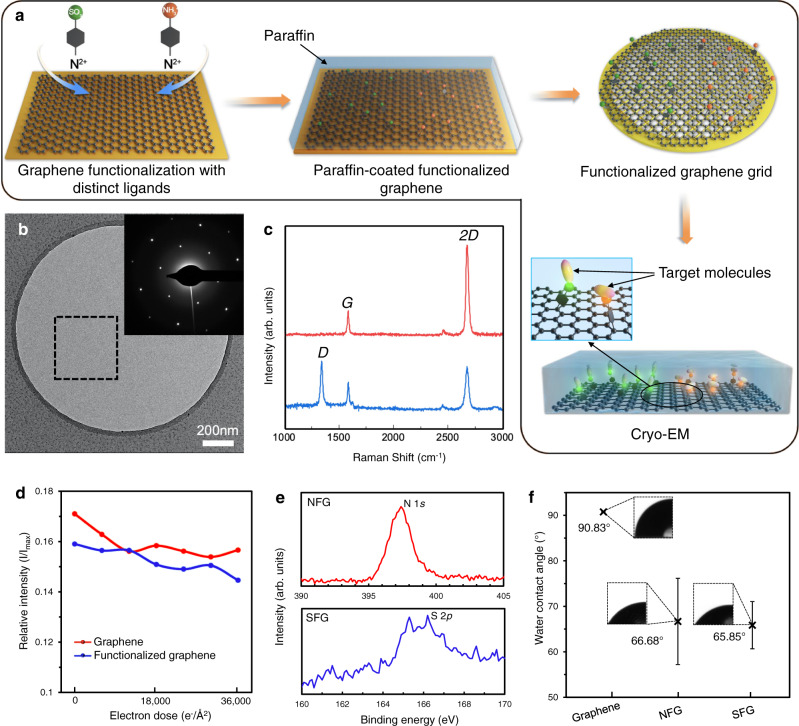
Characterization of the functionalized graphene grid. **a** Scheme of the functionalized graphene for cryo-EM analysis. **b** A representative TEM micrograph of the suspended functionalized graphene membrane. The inset is the diffraction pattern of the selected area (indicated by the dotted box). **c** Raman spectra of graphene (red) and functionalized graphene (blue). **d** The relative intensities of graphene (red) and functionalized graphene (blue), defined by the ratio of third-order integrated Bragg intensity (I) over the first-order integrated Bragg intensity (I_max_), are plotted with the accumulated electron dose. **e** X-ray photoelectron spectra of NFG (upper, red curve) and SFG (down, blue curve). **f** Water contact angles (WAC) of graphene, NFG and SFG. The averaged mean WAC (3 times independent measurements for graphene, 4 times independent measurements for NFG and 4 times independent measurements for SFG) are labelled, and error bars represent the standard deviations. NFG: NH_3_^+^-functionalized graphene; SFG: SO_3_^-^-functionalized graphene. Source data are provided as a Source Data file.

Based on the paraffin-aided transfer method, we further developed a procedure to functionalize the freshly-grown graphene before transferring onto EM grids to prevent potential contamination’s effect on the functionalization reaction (Fig. 2a). We synthesized two diazonium salts (Supplementary Fig. 4 and Supplementary Fig. 5): 4-aminobenzenediazonium tetrafluoroborate and 4-diazoniobenzenesulfonate tetrafluoroborate, containing functional groups of different electrostatic charges in physiological pH range (i.e., amino-group in the former and sulfa-group in the latter), and applied the dediazoniation reaction to introduce the functional groups onto the freshly-grown graphene surface. After different attempts and optimizations, the functionalization step proceeded effectively under moderate conditions (see Methods). Briefly, we incubated graphene with the diazonium salt solution (4-aminobenzenediazonium tetrafluoroborate in DMSO or 4-diazoniobenzenesulfonate tetrafluoroborate in deionized water) for 30 min at 40 °C. The covalently modified graphene was then transferred onto EM grids assisted by paraffin (Fig. 2b, and Supplementary Fig. 6). Such a graphene grid showed sharp diffraction spots, indicating that the dediazoniation reaction still preserved the overall graphene crystal lattice, although a *D* peak (1350 cm^−1^) was found on the Raman spectra of the functionalized graphene, suggesting the presence of the modification-induced defects (Fig. 2c). To evaluate the influence of the defects on the electron-radiation-resistance performance of graphene film, we measured and plotted the relative intensities of integrated Bragg reflections of non-functionalized and functionalized graphene, respectively (Fig. 2d). Both of them decayed with the accumulation of electron radiation in a similar rate up to a dose over 30,000 e^-^/Å^2^ (Fig. 2d), demonstrating the high stability of functionalized graphene under high-energy electron beam.

We further employed X-ray photoelectron spectroscopy (XPS) to characterize the elemental composition of the functionalized graphene and found the presence of N 1 *s* (Fig. 2e, upper) and S 2*p* (Fig. 2e, down) signals that indicated the successful bonded NH_3_^+^ and SO_3_^-^ groups to the graphene film, respectively. The functionalized graphene grids also exhibited higher hydrophilicity compared to the untreated graphene, as revealed by the water contact angle characterization. Graphene without functionalization treatment was relatively hydrophobic, whose water contact angle was ~91°, while the NH_3_^+^-functionalized graphene (NFG) and SO_3_^-^-functionalized graphene (SFG) grids have decreased water contact angles of ~67° and ~66°, respectively (Fig. 2f), allowing us to use them for cryo-EM specimen preparation (see Methods).

### Graphene with various charges to alter the orientational distribution of target particles

**Fig. 3:**
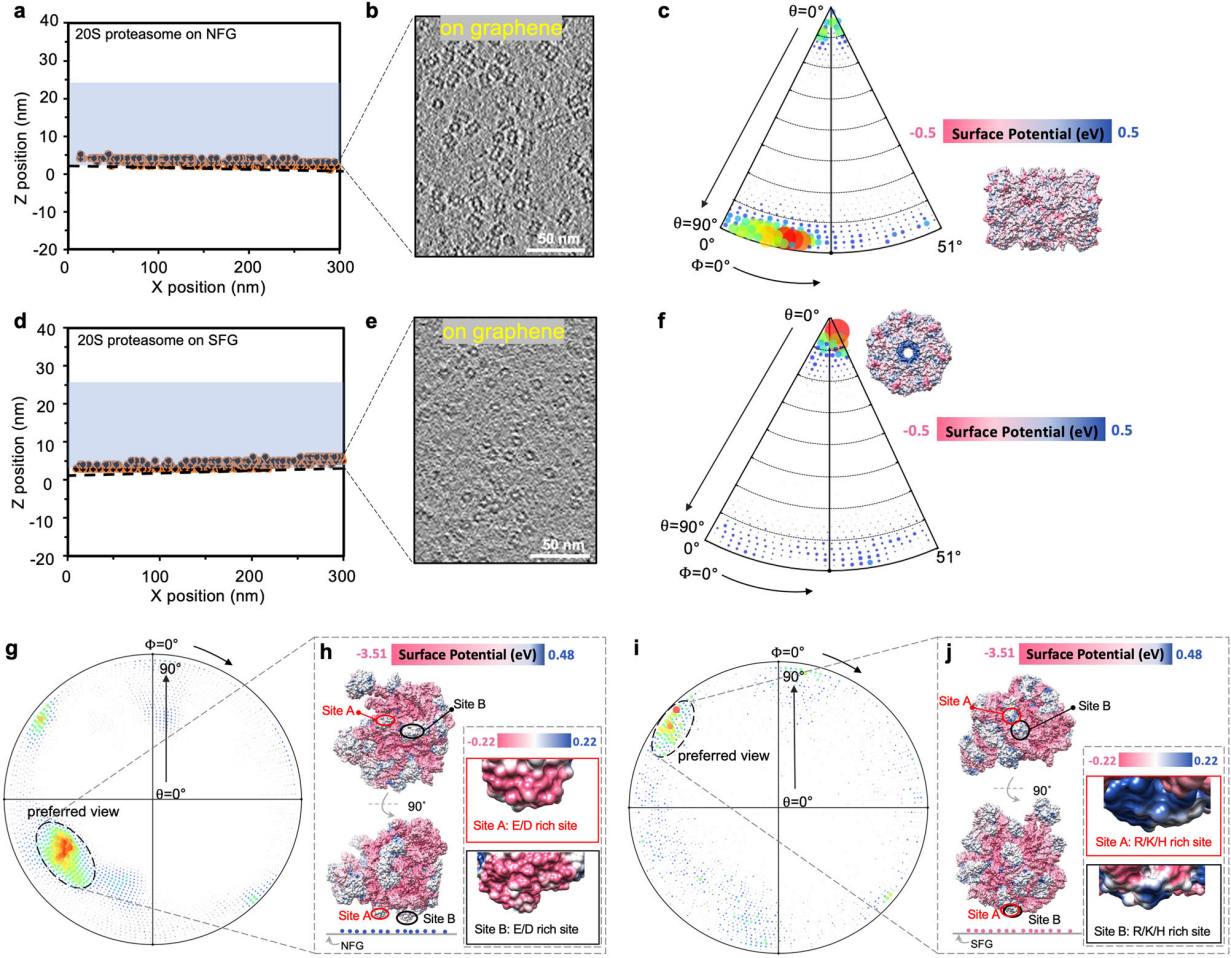
Graphene modified by distinct ligands for cryo-EM analysis. **a** The distribution of 20S proteasome particles on NFG membrane, revealed by cryo-ET reconstruction. Each spot indicates one individual particle. The black dotted line represents the graphene support. **b** The graphene surface section extracted from the cryo-tomogram of NFG-supported specimen, where the majority particles are distributed. **c** The Euler angle distribution of 20S proteasome particles on NFG. The bigger (redder) the spot, the more particles are in the corresponding orientation. The preferred view (side view) is displayed aside, with its map surface colored by electric potential. **d** The distribution of 20S proteasome particles on SFG membrane, revealed by cryo-ET reconstruction. **e** The graphene surface section extracted from the cryo-tomogram of SFG-supported specimen, where the majority particles are distributed. **f** The Euler angle distribution of 20S proteasome particles on SFG. The preferred view (top view) is displayed aside, with its map surface colored by electric potential. **g** The Euler angle distribution of 50S ribosome particles on NFG. **h** The preferred view of 50S ribosome on NFG. Upper: observed from perpendicular-to-graphene view. Down: observed from parallel-to-graphene view. The interacting sites, labelled as Site A (in red) and Site B (in black), are shown in more detail in the zoomed-in insets. **i** The Euler angle distribution of 50S ribosome on SFG. **j** The preferred view of 50S ribosome on SFG with similar setup as in (**h**). NFG: NH_3_^+^-functionalized graphene; SFG: SO_3_^-^-functionalized graphene. Source data are provided as a Source Data file.

We used the charged graphene grids to make cryo-EM specimens of 20S proteasome (Supplementary Fig. 7) and performed cryo-EM analysis of the complex (Fig. 3a–f). 20S proteasome displayed monodispersed particles on the charged graphene grids with high contrast, allowing us to perform 3D reconstruction of the complex. We first reconstructed the cryo-electron tomogram of these graphene-supported specimens and found that almost all the particles were absorbed onto the functionalized graphene surface, away from the AWI (Fig. 3a, d, and Supplementary Fig. 8a, b). Strikingly, 20S proteasome particles adopted different preferred projection views on the NFG (Fig. 3b) and SFG (Fig. 3e) surfaces. More specifically, 20S proteasome side view (rectangle shape) dominated on the NFG in comparison with more 20S proteasome top view (circle shape) on SFG, as revealed by the sections on the graphene surface extracted from the tomograms of NFG- and SFG-supported specimens. As controls, we also reconstructed the tomograms of 20S proteasome and ribosome on graphene treated by residual air glow discharge, both demonstrating mainly two-layer distribution of target particles: the majority close to the graphene surface and few on the AWI (Supplementary Fig. 8c, d). Note that, almost all the 20S proteasome particles on the air-glow treated graphene adopted side views on the graphene surface.

We further collected single-particle cryo-EM datasets and carefully analyzed the angular distribution of 20S proteasome on NFG and SFG and found varied orientation distribution of 20S proteasome on NFG (Fig. 3c) and SFG (Fig. 3f), consistent with the corresponding cryo-tomogram results. To eliminate any potential unknown factors that may bias the orientation variation, we also co-functionalized the two electrically opposite groups on one single graphene grid to characterize the orientation distribution of 20S proteasome from one droplet on the grid. As expected, we got the same result that side views dominated on the NH_3_^+^ modified region while top views dominated on SO_3_^-^ modified region (Supplementary Fig. 9). 20S proteasome has been frequently found to take either top view or side view, but not both together, on conventional grids as well as the graphene grids. What we found here now suggests the feasibility to solve the preferential orientation problem of macromolecule complexes on one graphene grid with two charged functional groups.

We next applied the two kinds of charged graphene grids to cryo-EM analysis of 50S ribosome (Fig. 3g–j, and Supplementary Fig. 10). The preferred view of 50S ribosome (labeled in Fig. 3g) on the NFG was basically absent on the SFG (Fig. 3i). We further coordinated the view distribution with the electrostatic potential distribution on the surface of a 50S ribosome molecule. The dominate view of 50S ribosome on the NFG was likely attributed to the electrostatic interaction between the positively charged groups of NFG and the two negatively charged sites on the ribosome surface (Fig. 3h, Site A: E86, D91, E136, E143, E144, E115 in chain L; and Site B: E2, D7, E16, E122, E127, D140, E144, E152, D154, E155, D191, E197, E198, in chain E). And the preferred view on SFG can be deduced through the interaction between the negatively charged groups of SFG and two positively charged sites on the ribosome surface (Fig. 3j, Site A: K31, K36, R49, R51, K52, in chain 3 and H60 in chain S; and Site B: H31, K56, R63, R69, K78, R86, R90, R96, R118, K121 in chain N). Such an interaction with the predictable orientation of macromolecules on the charged graphene surface implicates a potential application to specifically anchor macromolecules with designed orientation for other research purposes such as single molecule monitoring of a translational process in a controlled ribosome orientation.

### Graphene with various charges to solve the preferential orientation problem of L1.LtrB RNP

**Fig. 4:**
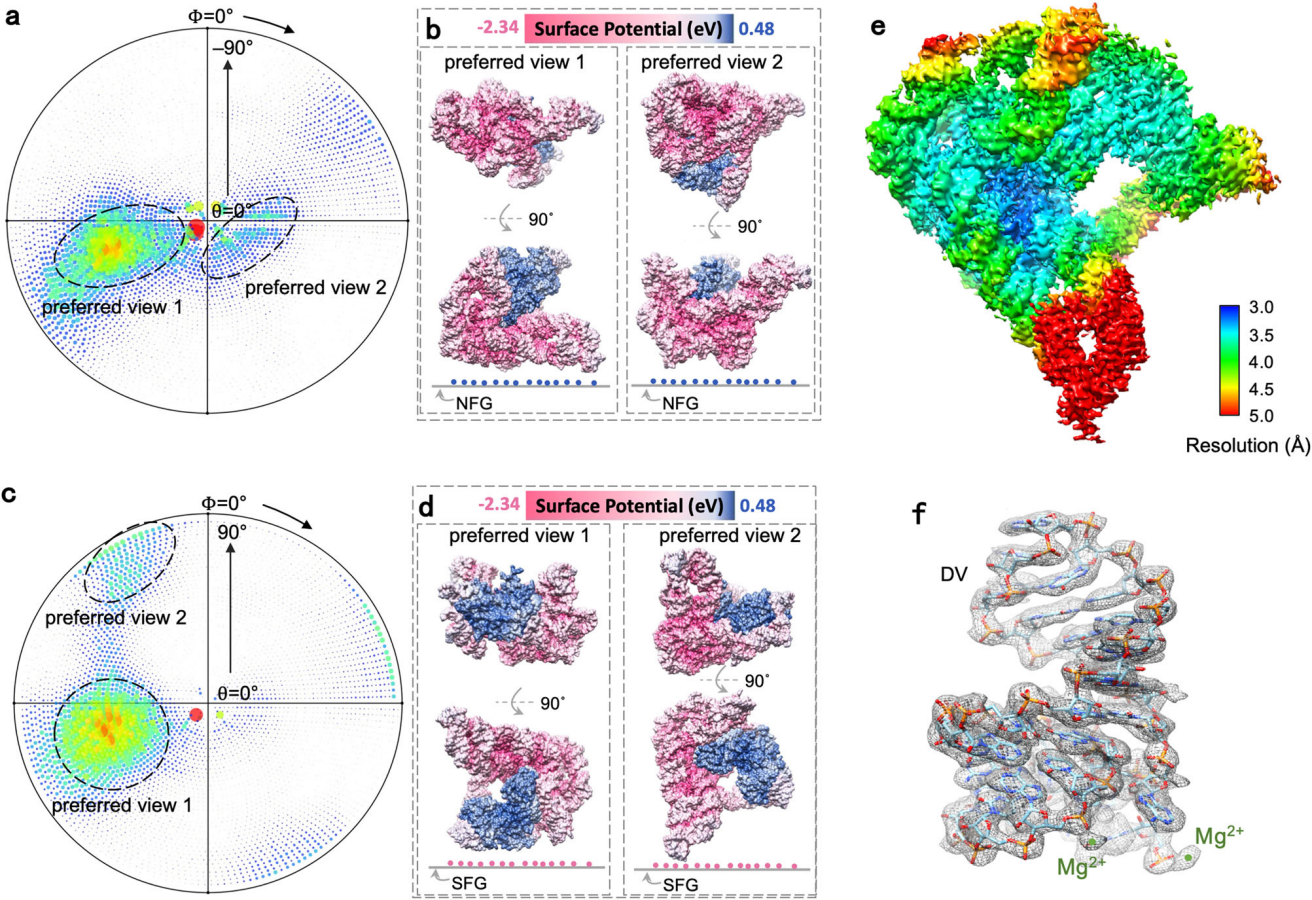
Graphene modified by distinct ligands for cryo-EM reconstruction of Ll.LtrB RNP. **a–d** The particle-orientation distribution of L1.LtrB RNP complex on NFG (**a**, **b**) and SFG (**c**, **d**). Note that the preferred view 1 in (**a**) is the opposite-side projection of the preferred view 1 in (**c**). **e** The reconstructed 3D density map of L1.LtrB RNP complex using the combined particles from NFG and SFG, colored by local resolution estimation. **f** The density of DV of L1.LtrB RNP with the corresponding model docked (PDB: 8H2H). NFG: NH_3_^+^-functionalized graphene; SFG: SO_3_^-^-functionalized graphene. Source data are provided as a Source Data file.

We lastly applied the charged graphene grids to the group IIA intron RNP complex, L1.LtrB in *Lactococcus lactis*, which is composed of an RNA chain and a polypeptide chain. The cryo-EM structure determination of L1.LtrB RNP was largely impaired by the notorious preferred orientation problem on either holey carbon films or conventional supporting films^17,18^. We used the NFG and SFG to prepare the cryo-EM specimen of Ll.LtrB RNP (Supplementary Fig. 11) and found various views on the NFG (Fig. 4a, b) and SFG (Fig. 4c, d), in contrast to only one dominant view on conventional graphene treated by residual air-glow discharge (Supplementary Fig. 12). The analysis of the orientational distribution showed that the negatively charged RNA chain plays a key role when interacting with the NFG (Fig. 4b) while the polypeptide chain with relatively positive charges directs the interaction with the SFG (Fig. 4d).

The other notable thing is that we picked 972,269 particles from air-glow-treated conventional graphene support (Supplementary Fig. 12) but only 55,783 particles were kept for the final 3D reconstruction. This means that the useful particle proportion on the conventional graphene support is 5.7%, much lower than that on SFG (35.7%) or NFG (23.5%), indicating a more friendly interacting interface with Ll.LtrB RNP particles on our modified graphene.

By using a combination of particle images from the two differently charged graphene grids, we were able to obtain the reconstruction of Ll.LtrB RNP complex at 3.2-Å resolution (Fig. 4e). This marked the highest resolution of the endogenously purified group II intron RNP, where the bases and metal ions in the catalytic center can be clearly recognized (Fig. 4f). The solution of the structure was so straightforward that we did not bother the orientation problem or denaturation problems at all during the 3D reconstruction process. In comparison with our previous effort on conventional thin-carbon film support, we used here 399,660 particle images out of 2757 micrographs (1285 micrographs from NFG and 1472 from SFG) to achieve this high resolution (Supplementary Table 1 and Supplementary Table 2), proving the robustness of the functionalized grids with charges.

## Discussion

In summary, we developed a robust clean transfer strategy to fabricate charged graphene EM grids using paraffin as the transfer mediator. The graphene membrane was functionalized with various electrostatic properties by effective dediazoniation reactions. Our strategy provided a solution with the following considerations: 1) The electrostatic interaction is much stronger, compared with other interaction modes such as the van der Waals force, allowing us to effectively manipulate the orientational distribution of target macromolecules. 2) We adopted a dediazoniation reaction to functionalize graphene in moderate conditions with high efficiency. 3) Graphene was modified prior to its transfer onto EM grids to reduce the chance of contamination and then transferred to EM grids with a paraffin-assistant graphene grid fabrication method.

The charged graphene grids provide different surfaces for protein molecules or macromolecule complexes to interact with, based on the target molecule’s surface charge properties, therefore allowing multiple orientations on various charged graphene grids and avoiding impairment by the AWI.

## Methods

### Synthesis of diazonium salts

0.22-gram p-phenylenediamine and 0.35-gram p-aminobenzene sulfonic acid were dissolved in 5 ml deionized water, and then added by 1 ml 50% HBF_4_, and the mixture was stirred at 0 °C for 5 min. 1 ml 2.2 M sodium nitrite solution was added into the mixture at 0 °C and the solution was stirred vigorously for 1 hr followed by filtration to obtain a crude product. We used acetone to dissolve the solid, used ether to precipitate the product, and finally filtered the product again to get pure diazonium salt. The benzene-sulfonic acid group diazonium salt was dissolved in deionized water and benzene-anilinic group diazonium salt was dissolved in DMSO, both at a final concentration of 20 mM. For better reaction efficiency, 20 µl 1% (by wt) anionic surfactant SDS aqueous solution was added into 100 µl 20 mM diazonium salt solution to form the reactant solution for later graphene functionalization procedures.

### NMR characterization of diazonium salts

The ^1^H NMR spectra were recorded at room temperature on a Bruker AVANCE^III^ 400 MHz spectrometer in D_2_O (for 4-diazoniobenzenesulfonate tetrafluoroborate) or DMSO-d_6_ (for 4-aminobenzenediazonium tetrafluoroborate), using TMS or solvent peak as a standard. Low-resolution mass spectral analyses were performed with Waters AQUITY UPLC^TM^/MS.

### Functionalization of graphene membrane

Graphene was grown on copper foil by the chemical vapor deposition (CVD) method. We firstly cut a piece of graphene-coated copper foil and dropped the diazonium salt solution onto graphene surface, followed by a 30-min incubation at 40 °C. Afterwards, graphene membrane was washed by 1 ml deionized water for 5 times and immersed into deionized water for an additional 0.5–1 h to ensure the complete removal of residual diazonium reagent from graphene surface.

### Transfer of functionalized graphene membrane on EM grids

We firstly removed the unfunctionalized graphene grown on the backside of the copper foil by placing the foil in a Gatan plasma machine (Model 950) with its backside up, and performed the plasma treatment at a O_2_ (11.5 sccm) and Ar (35 sccm) atmosphere for 30 s. We then heated the copper foil to 65 °C, and added a small piece of paraffin (Sigma-Aldrich 18634) onto its functionalized graphene side, which immediately melted and spread to cover the whole foil. After the paraffin spread as a thin film on top of the graphene, the copper foil was cooled down to room temperature and the paraffin solidified. Afterwards, the paraffin-graphene-copper was gently put on the surface of 0.5 M ammonium persulfate (APS) solution with the paraffin side facing up, and incubated for 5 h. After the copper foil was thoroughly etched off, the paraffin-graphene was rinsed with deionized water several times, then transferred into a 40 °C incubator and incubated for at least 1 h, to stretch and diminish wrinkles in the graphene membrane. After that, we used Quantifoil grids (Au 300 mesh, R 1.2/1.3) to scoop out the paraffin-coated graphene from the 40 °C deionized water and air-dried the graphene grids in a clean chamber at 40 °C overnight. The paraffin-graphene-grids supported by oil-absorbing paper (The Mentholatum company, B00004Y) were then incubated at 65 °C for 5–10 min to remove most paraffin coating. Afterwards, the grid was soaked into petroleum ether (preheated to 65 °C) and stirred at 60 rpm for 3 h to completely remove the paraffin. Finally, the grids were washed by acetone, isopropyl alcohol (IPA) and water consecutively to clean off residual petroleum ether and air-dried.

### Characterization of the cleanliness of graphene membrane

We used graphene membrane transferred by PMMA-assisted method^15^ as control, to compare and demonstrate the cleanliness of graphene grid fabricated in this work. To better describe the cleanliness of graphene in the holes on EM grid, here we define a hole with more than 5 contamination spots with the area of >400 nm^2^ as highly contaminated hole, and we found almost all of the holes on the graphene grids fabricated by PMMA-assisted transfer method were highly contaminated. For graphene grid fabricated by paraffin-assisted method, we imaged 141 holes and found 123 holes (~87%) with no or no more than five contamination spots, and 98 holes (~70%) with no or no more than three contamination spots.

### Characterization of the functionalized graphene membrane

X-ray photoelectron spectra was collected on a ESCALAB Xi + (ThermoFisher Scientific), with the monochromatic Al Kα X-ray beam spot of 500 μm. Raman spectra was measured on a LabRAM HR-800 (Horiba) with the 633-nm laser wavelength. For water contact angle measurement, we applied 0.7 μl deionized water onto the grid surface and used OCA15Pro (Dataphysics) to collect the water contact angles. The roughness of the suspended graphene surface was characterized using AFM (Oxford Instruments Asylum Research, Cypher VRS).

### Biological samples preparation

*Thermoplasma acidophilum* 20S proteasome was expressed and purified from *Escherichia coli* BL21(DE3) strain by following previously reported protocols^19^. Briefly, we transformed the plasmids into *Escherichia coli* competent cells, which contained α and β subunits of 20S proteasome, with the N terminal of β subunit tagged by six histidines. After induced expression, *Escherichia coli* cells were sonicated and the cell lysates were then applied onto affinity nickel column to get purified 20S proteasome sample.

Ll.LtrB RNP were purified from *Lactococcus lactis* strain IL1403 according to the published methods^17^. We firstly constructed a plasmid containing Ll.LtrB intron with the ORF region in its DIV depleted, its encoded protein LtrA and a chitin binding domain (CBD), which was then transformed into *Lactococcus lactis* strain IL1403 and allowed for induction. The cell lysates were loaded onto affinity chitin column, followed by dithiothreitol elution and ultracentrifugation to get purified Ll.LtrB RNP sample.

*E. Coli* ribosome was purchased from NEB company (P0763S).

### Cryo-EM specimen preparation

For sample vitrification, we firstly pipetted 7 μl sample buffer (100 mM Tris-HCl pH 8.0, 150 mM NaCl) onto the functionalized graphene grids, and incubated the grids for 2 min at a chamber of high humidity. Afterwards, the grids were edge-blotted by filter papers to remove the extra buffer, immediately followed by adding 4-μl sample solution, incubated for 2 min and then transferred into Vitrobot Mark IV (ThermoFisher Scientific). The humidity of Vitrobot chamber was set as 100%, and the temperature as 8 °C. The grids were blotted by filter papers (Ted Pella) for 1 s with a force of −2, and then plunge-frozen into liquid ethane. The vitrified specimens were stored in liquid nitrogen. To prepare conventional graphene-supported cryo-specimen, we pipetted 4 μl sample solution onto freshly glow-discharged graphene grid, and blotted the grid for 1~2 s with a force of −2, using Vitrobot Mark IV (ThermoFisher Scientific). After blotting, the grid was frozen into liquid ethane and stored in liquid nitrogen.

### Single-particle cryo-EM data collection and analysis

The cryo-EM datasets were collected on a Titan Krios (300 kV) microscope equipped with a Gatan K3 Summit direct electron detector. The pixel size was 0.97 Å and every movie contained 32 frames with a summed dose of 50 e^-^/Å^2^. The movies were motion-corrected by MotionCor2^20^. The CTF values were estimated by CTFFIND4^21^ and the resulted micrographs were imported to Relion3.1 for particle picking and 2D classification^22^. After discarding junk particles grouped in bad 2D classes, the remaining particles were imported for 3D classification and refinement, to obtain the final 3D reconstructions. The final particle number used for the reconstruction of Ll.LtrB was 399,660, and the resolution was 3.2 Å, estimated by the Fourier Shell Correction (FSC) = 0.143 cutoff criteria. Structural visualization and analysis were performed in UCSF Chimera^23^.

### Cryo-ET data collection and analysis

Tilted series were acquired on a Titan Krios (300 kV) microscope equipped with a Gatan K3 Summit direct electron detector, from −60° to +60° with a step of 3° using SerialEM^24^. We collected 8 frames at each tilt angle, with a summed dose of 3 e^-^/Å^2^, which were further motion-corrected by MotionCor2^20^. We used IMOD software^25^ to align and reconstruct the tomograms.

### Reporting summary

Further information on research design is available in the Nature Portfolio Reporting Summary linked to this article.

## Supplementary information

Supplementary Information

Reporting Summary

## Data Availability

Data supporting the findings in this manuscript are available from the corresponding authors upon requests. The density map has been deposited in the Electron Microscopy Data Bank (EMDB) under accession number EMD-33039 (Ll.LtrB). The atomic coordinates have been deposited in the Protein Data Bank (PDB) under accession number 8H2H (Ll.LtrB). The source data underlying Fig. 1c, Fig. 2d–f, Fig. 3a, c, d, f, g, and i and, Fig. 4a, c, and Supplementary Fig. 3, Supplementary Fig. 9d–f, Supplementary Fig. 11 and Supplementary Fig. 12b are provided as a Source Data file. Source data are provided with this paper.

## Source data

Source Data

## References

[1] Cheng, Y. F. Single-particle cryo-EM-How did it get here and where will it go. *Science* **361**, 876–880 (2018). 10.1126/science.aat4346 PMC6460916 30166484

[2] Glaeser RM (2021). Preparing Better Samples for Cryo-Electron Microscopy: Biochemical Challenges Do Not End with Isolation and Purification. Annu Rev. Biochem.

[3] Tan YZ (2017). Addressing preferred specimen orientation in single-particle cryo-EM through tilting. Nat. Methods.

[4] Han BG (2016). Long shelf-life streptavidin support-films suitable for electron microscopy of biological macromolecules. J. Struct. Biol..

[5] Han Y (2020). High-yield monolayer graphene grids for near-atomic resolution cryoelectron microscopy. Proc. Natl Acad. Sci. USA.

[6] Liu N (2019). Bioactive Functionalized Monolayer Graphene for High-Resolution Cryo-Electron Microscopy. J. Am. Chem. Soc..

[7] Palovcak E (2018). A simple and robust procedure for preparing graphene-oxide cryo-EM grids. J. Struct. Biol..

[8] Russo CJ, Passmore LA (2014). Controlling protein adsorption on graphene for cryo-EM using low-energy hydrogen plasmas. Nat. Methods.

[9] Zheng L (2020). Robust ultraclean atomically thin membranes for atomic-resolution electron microscopy. Nat. Commun..

[10] Geim AK, Novoselov KS (2007). The rise of graphene. Nat. Mater..

[11] Nan L (2021). Reduced graphene oxide membrane as supporting film for high-resolution cryo-EM. Biophysics Rep..

[12] Naydenova K, Peet MJ, Russo CJ (2019). Multifunctional graphene supports for electron cryomicroscopy. Proc. Natl Acad. Sci. USA.

[13] Wang, F. et al. Amino and PEG-amino graphene oxide grids enrich and protect samples for high-resolution single particle cryo-electron microscopy. *J. Structural Biol.* **209**, 107437 (2020). 10.1016/j.jsb.2019.107437 PMC7272056 31866389

[14] D’Imprima, E. et al. Protein denaturation at the air-water interface and how to prevent it. *Elife* **8**, e42747 (2019). 10.7554/eLife.42747 PMC6443348 30932812

[15] Leong WS (2019). Paraffin-enabled graphene transfer. Nat. Commun..

[16] Zhang J (2020). New Growth Frontier: Superclean Graphene. ACS Nano.

[17] Qu G (2016). Structure of a group II intron in complex with its reverse transcriptase. Nat. Struct. Mol. Biol..

[18] Liu N (2020). Exon and protein positioning in a pre-catalytic group II intron RNP primed for splicing. Nucleic Acids Res..

[19] Li X (2013). Electron counting and beam-induced motion correction enable near-atomic-resolution single-particle cryo-EM. Nat. Methods.

[20] Zheng SQ (2017). MotionCor2: anisotropic correction of beam-induced motion for improved cryo-electron microscopy. Nat. Methods.

[21] Rohou A, Grigorieff N (2015). CTFFIND4: Fast and accurate defocus estimation from electron micrographs. J. Struct. Biol..

[22] Scheres SH (2012). RELION: implementation of a Bayesian approach to cryo-EM structure determination. J. Struct. Biol..

[23] Pettersen EF (2004). UCSF chimera - A visualization system for exploratory research and analysis. J. Comput Chem..

[24] Mastronarde DN (2005). Automated electron microscope tomography using robust prediction of specimen movements. J. Struct. Biol..

[25] Kremer JR, Mastronarde DN, McIntosh JR (1996). Computer visualization of three-dimensional image data using IMOD. J. Struct. Biol..

