# Supplementary material for: Functionalized graphene grids with various charges for single-particle cryo-EM
Source: Nat Commun. 2022 Nov 7;13:6718. doi: 10.1038/s41467-022-34579-w (PMC9640669; doi:10.1038/s41467-022-34579-w)
Supplement: Supplementary file 1 — Supplementary Information [file 41467_2022_34579_MOESM1_ESM.docx]

**Supplementary Information**

**Functionalized graphene grids with various charges for single-particle cryo-EM**

Ye Lu^1,2,3,4^, Nan Liu^1,3,4^^*^, Yongbo Liu^3,4,5,6^, Liming Zheng^7,8^ Junhao Yang^1,3,4^, Jia Wang^1,3,4^, Xia Jia^1,2,3,4^, Qinru Zi^1,9^, Hailin Peng^7,8*^, Yu Rao^3,4,5,6*^, Hong-Wei Wang^1,2,^^3,4*^

^1^School of Life Sciences, Tsinghua University, Beijing 100084, China.

^2^Tsinghua-Peking Joint Center for Life Sciences, Tsinghua University, Beijing 100084, China.

^3^Beijing Advanced Innovation Center for Structural Biology, Beijing Frontier Research Center for Biological Structures, Tsinghua University, Beijing 100084, China.

^4^Ministry of Education Key Laboratory of Protein Sciences, Tsinghua University, Beijing 100084, China.

^5^Ministry of Education Key Laboratory of Bioorganic Phosphorus Chemistry & Chemical Biology, Tsinghua University, Beijing 100084, China.

^6^School of Pharmaceutical Sciences, Tsinghua University, Beijing, 100084, China.

^7^Center for Nanochemistry, Beijing Science and Engineering Center for Nanocarbons, Beijing National Laboratory for Molecular Sciences, College of Chemistry and Molecular Engineering, Peking University, Beijing 100871, China.

^8^Beijing Graphene Institute, Beijing 100095, China.

^9^College of Life Sciences, Wuhan University, Wuhan, Hubei, 430072, China.

These authors contributed equally to this work: Ye Lu, Nan Liu, Yongbo Liu,Liming Zheng

*Correspondence should be addressed to H.W: [hongweiwang@tsinghua.edu.cn](mailto:hongweiwang@tsinghua.edu.cn), H.P: [hlpeng@pku.edu.cn](mailto:hlpeng@pku.edu.cn); Y.R: yrao@tsinghua.edu.cn; or N.L: nanliuem@tsinghua.edu.cn


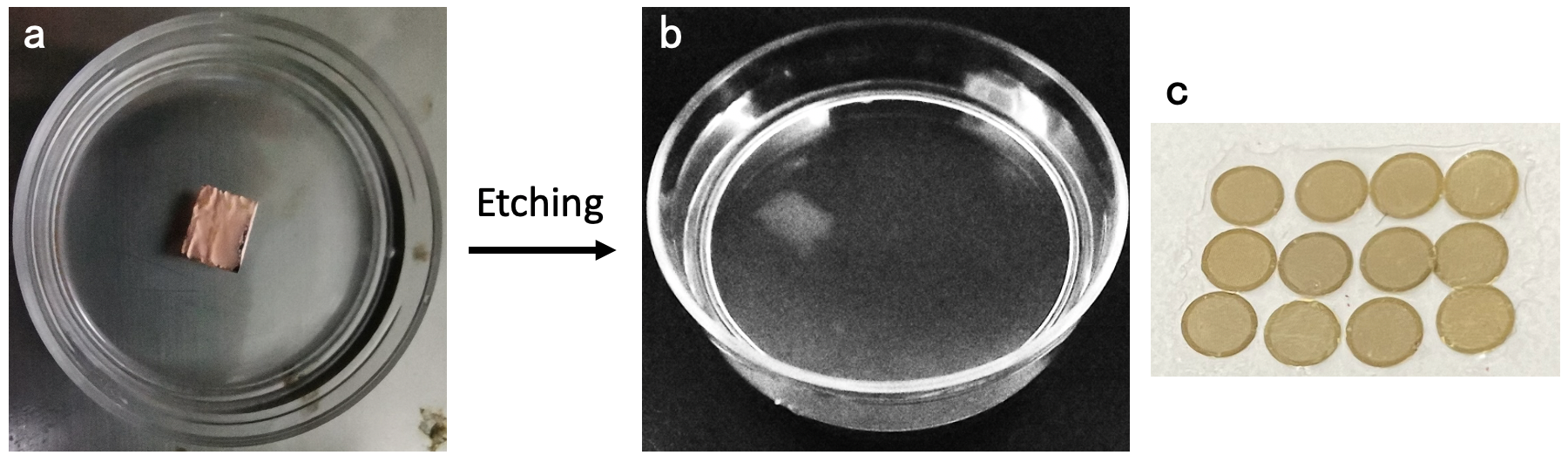


**Supplementary Fig. 1. Paraffin-assisted graphene transfer onto EM grids.** **a.** Paraffin-graphene-copper was floated on the surface of 0.5 M ammonium persulfate (APS) solution. **b.** After the copper was etched off, paraffin-graphene was rinsed by floating on the surface of deionized water. **c.** Paraffin-graphene was coated onto a dozen of EM grids.


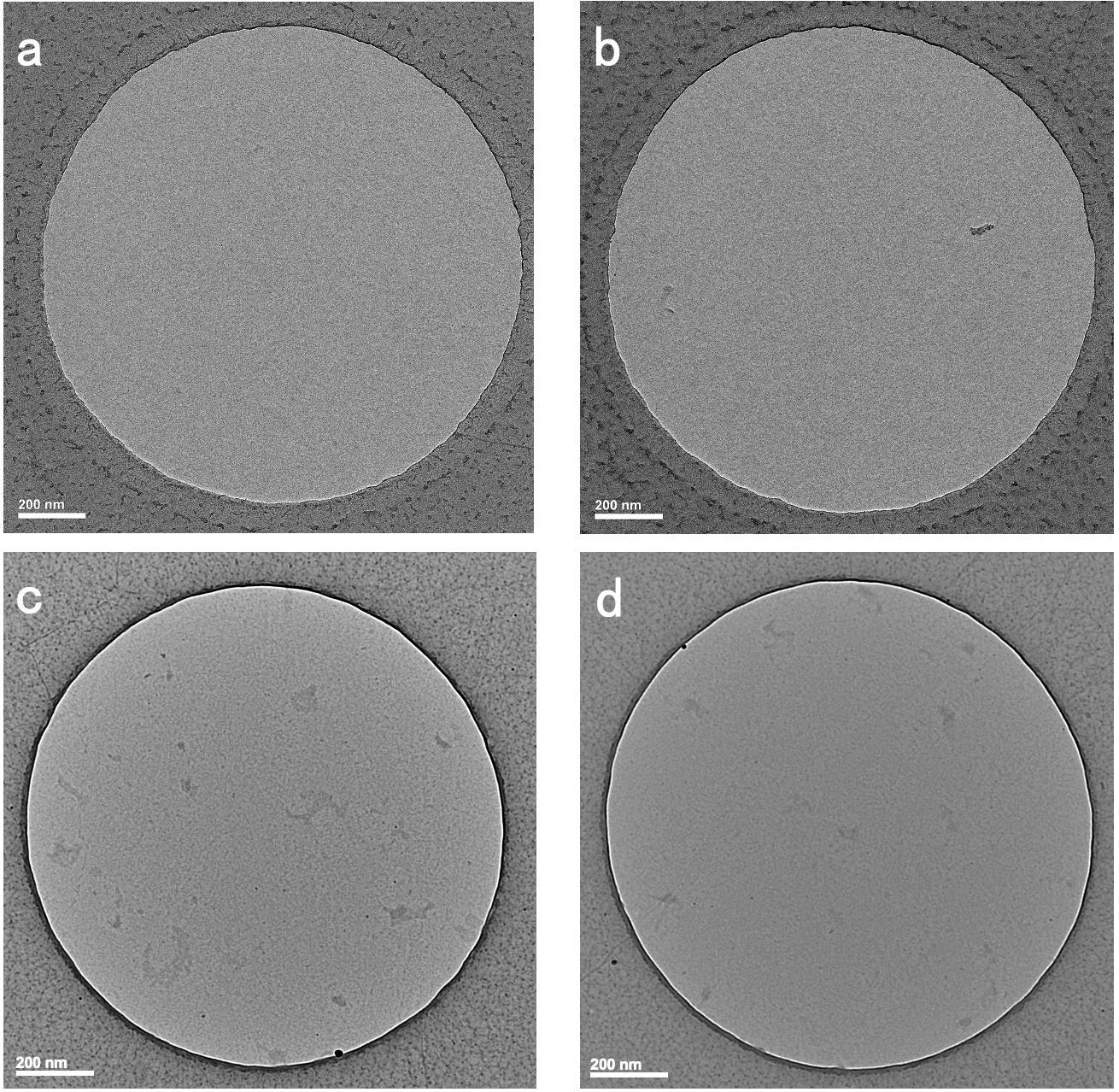


**Supplementary Fig. 2. TEM characterization of the graphene grids.** **a**-**b**. Representative TEM micrographs of graphene suspended on grid holes produced by paraffin-assisted graphene transfer method, 87% of which were with no or few contamination spots and supposed to be good regions for cryo-EM specimen preparation. Similar results in **a-b** have been repeated in 4 grids. **c**-**d**. Representative TEM images of graphene grid transferred by PMMA, where almost all the holes were visibly contaminated. Similar results in **c-d** have been repeated in 3 grids.

**Supplementary Fig. 3. AFM characterization of the paraffin-assisted transferred graphene grid.** A low-magnification AFM image. The hole with distinctly dark contrast indicated graphene-broken region. The AFM characterization of graphene grids transferred by paraffin-assisted method has been repeated three times. Source data are provided as a Source Data file.

^1^H NMR (400 MHz, DMSO-d6): δ (ppm) 8.32 (s, 2H), 8.14 (d, 2H), 6.81 (d, 2H); LRMS (ESI) calculated for C_6_H_5_N_4_^+^ [M]^+^: 120.13, found 120.01.

**Supplementary Fig. 4. NMR spectra of benzene-anilinic group diazonium salt.**

^1^H NMR (400 MHz, D_2_O): δ (ppm) 8.72 (d, 2H), 8.29 (d, 2H); LRMS (ESI) calculated for C_6_H_5_SO_3_N_2_^+^ [M]^+^: 185.18, found 185.01.

**Supplementary Fig. 5. NMR spectra of benzene-sulfonic acid group diazonium salt.**


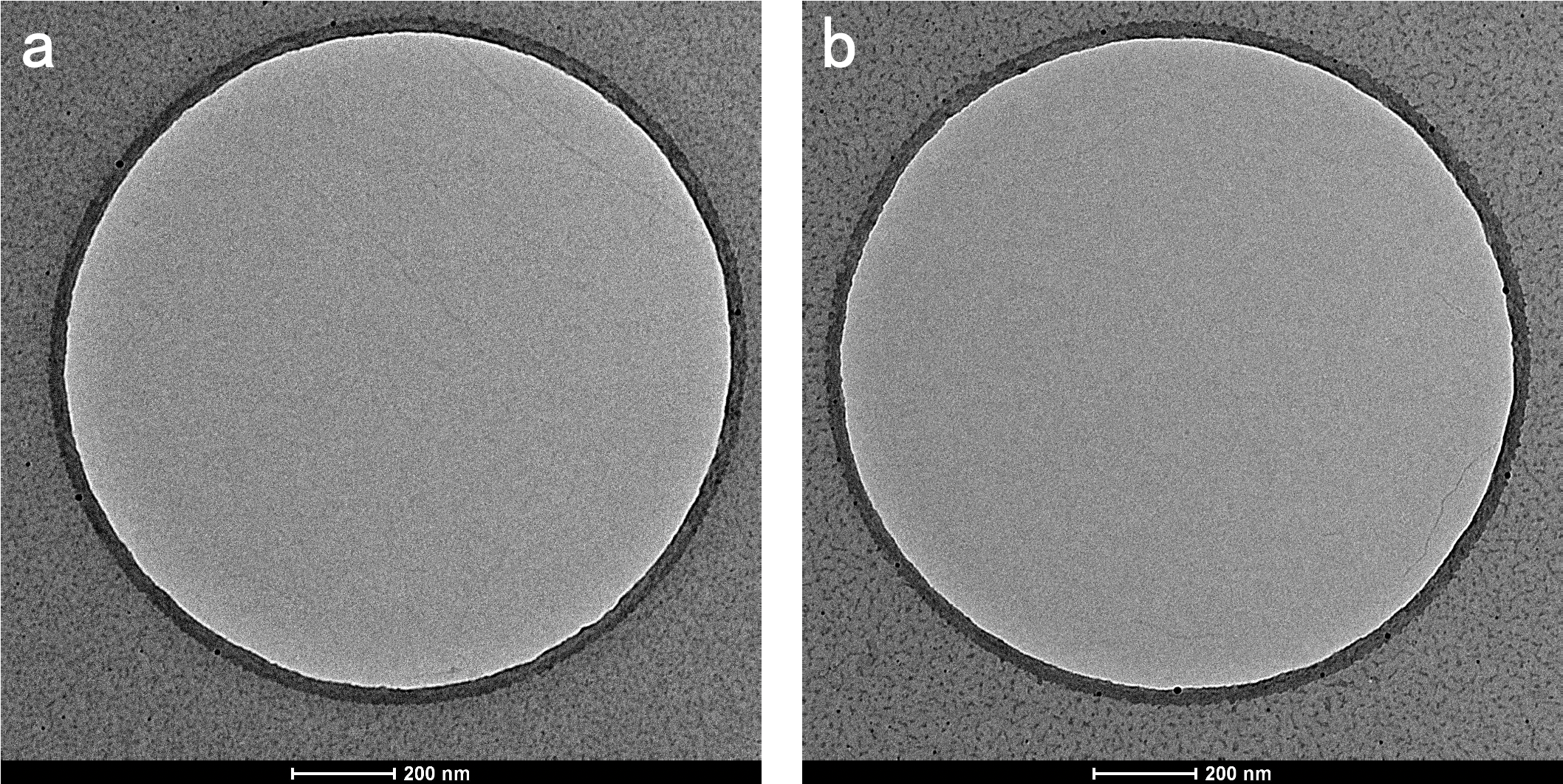


**Supplementary Fig. 6. High-magnification TEM characterization of NFG (a) and SFG (b) grids.** To make the graphene-covering holes more visible, these micrographs were taken at a defocus of -6 µm. The TEM characterization of both NFG and SFG has been repeated 8 times. NFG: NH_3_^+^-functionalized graphene; SFG: SO_3_^-^-functionalized graphene.


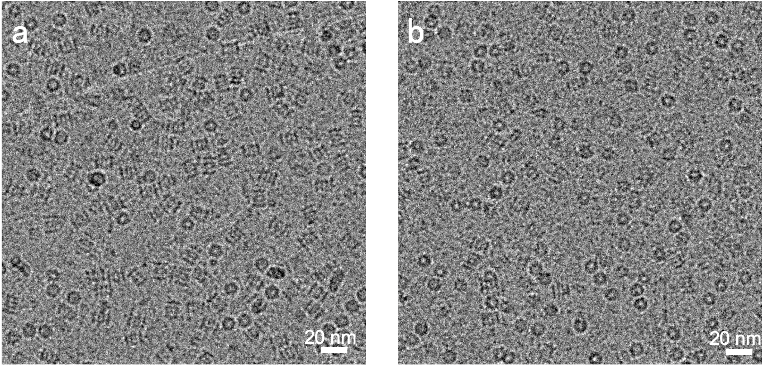


**Supplementary Fig. 7. Cryo-EM micrographs of 20S proteasome on NFG (a) and SFG (b) grids.** The ratio of top view over side view was higher on SFG (**a**) than that on NFG (**b**). 76 micrographs for 20S proteasome on NFG (**a**) and 61 micrographs for 20S proteasome on SFG (**b**) were collected for orientational distribution analysis. NFG: NH_3_^+^-functionalized graphene; SFG: SO_3_^-^-functionalized graphene.


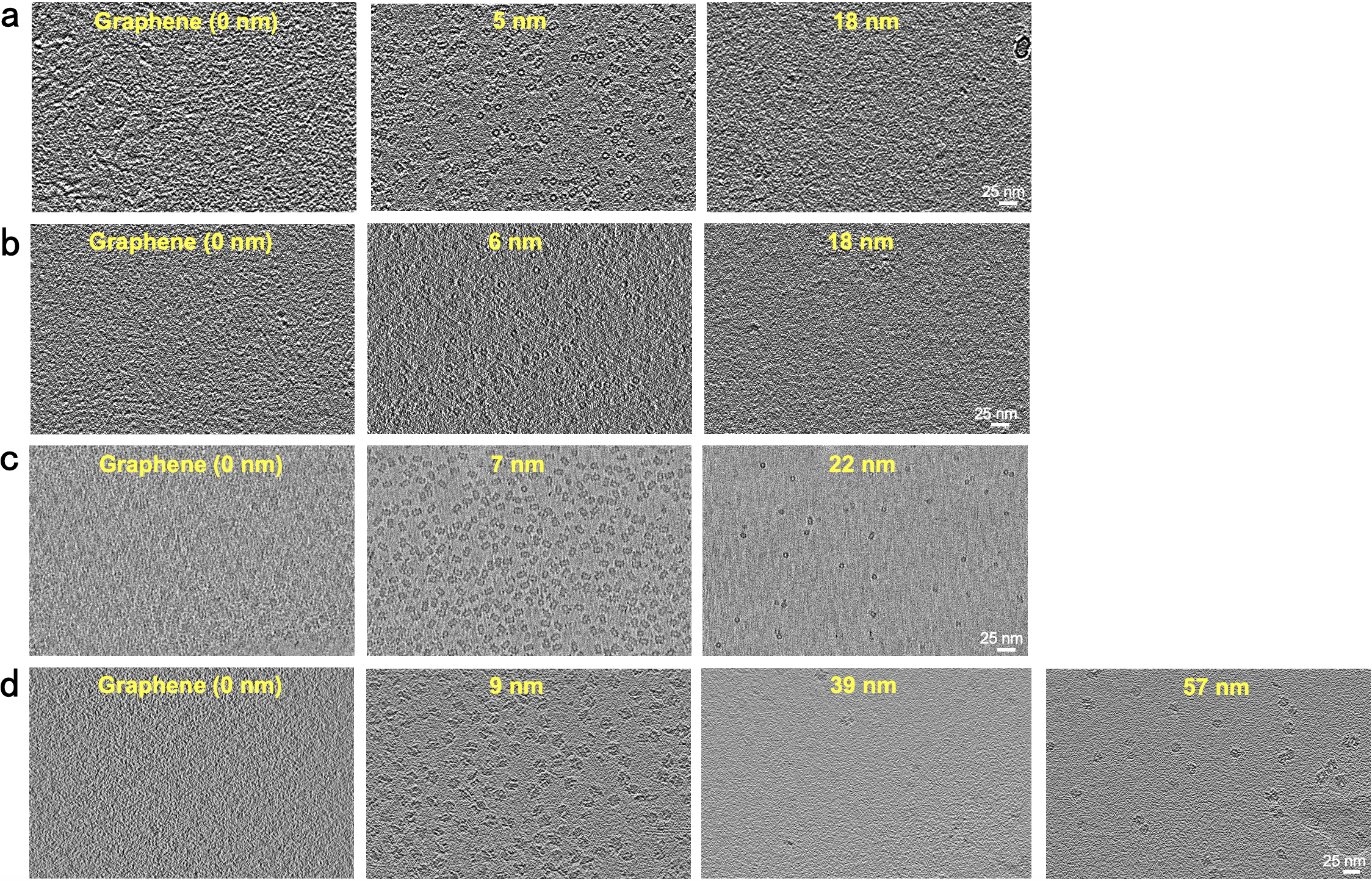


**Supplementary Fig. 8. Cryo-ET analysis of target particles on different graphene grids**. **a**-**c** 20S proteasome on NFG (**a**), SFG (**b**) and conventional graphene support (**c**). From left to right are graphene layer, close-to-graphene layer and close-to-air-water interface layer, extracted from the reconstructed tomograms of the cryo-specimens. **d**. Ribosome specimen on conventional graphene support. From left to right are graphene layer, close-to-graphene layer, middle-in-ice layer and close-to-air-water interface layer. Since the ice thickness of ribosome specimen is relatively larger than 20S proteasome and thereby we are able to characterize the particle distribution in the middle ice layer. The location of these layers relative to graphene support is labelled in yellow. Both (**c**) and (**d**) demonstrate mainly two-layer distribution of target particles: the majority close to graphene surface and few on the air-water interface. NFG: NH_3_^+^-functionalized graphene; SFG: SO_3_^-^-functionalized graphene.


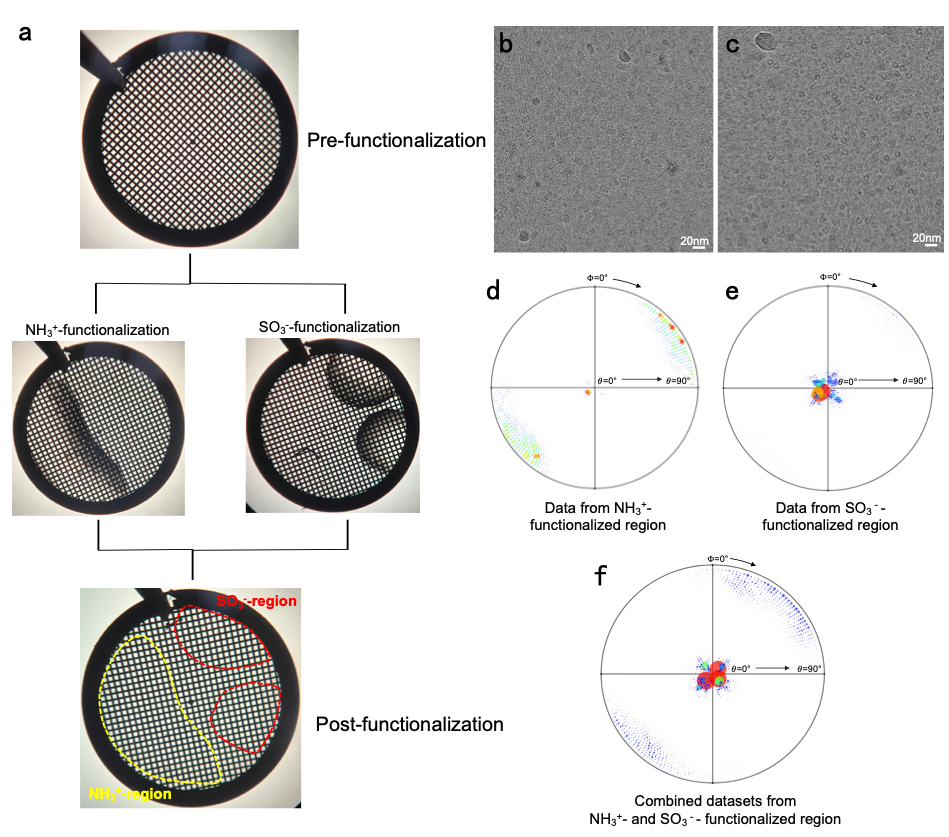


**Supplementary Fig. 9. Co-functionalization of graphene on one EM grid with different charge groups. a.** Optical images demonstrating that benzene-sulfonic and benzene-anilinic groups were co-functionalized onto different parts of the same graphene grid. The region circled by dotted yellow line in the post-functionalization grid indicates the benzene-anilinic group functionalized region, while the red lines circle the benzene-sulfonic region. **b.** A representative cryo-EM micrograph of 20S proteasome from NH_3_^+^-functionalized region. **c.** A representative cryo-EM micrograph of 20S proteasome from SO_3_^-^-functionalized region. 458 micrographs for 20S proteasome on NH_3_^+^-functionalized region (**b**) and 391 micrographs for 20S proteasome on SO_3_^-^-functionalized region (**c**) were collected for orientational distribution analysis. **d.** The Euler angle distribution of 20S proteasome on benzene-anilinic region. **e.** The Euler angle distribution of 20S proteasome on benzene-sulfonic region. **f.** The Euler angle distribution of the 20S proteasome datasets combined from both benzene-sulfonic and benzene-anilinic regions. The distribution is consistent with **Figure 3**. Source data are provided as a Source Data file.


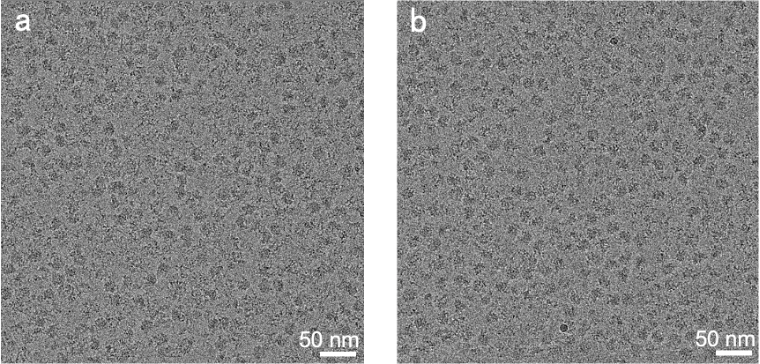


**Supplementary Fig. 10. Cryo-EM micrographs of ribosome on NFG (a) and SFG (b) grids.** 632 micrographs for ribosome on NFG (**a**) and 493 micrographs for ribosome on SFG (**b**) were collected for orientational distribution analysis. NFG: NH_3_^+^-functionalized graphene; SFG: SO_3_^-^-functionalized graphene.

**Supplementary Fig. 11. Workflow of cryo-EM reconstruction of Ll.LtrB RNP on NFG and SFG grids.** NFG: NH_3_^+^-functionalized graphene; SFG: SO_3_^-^-functionalized graphene. Source data are provided as a Source Data file.


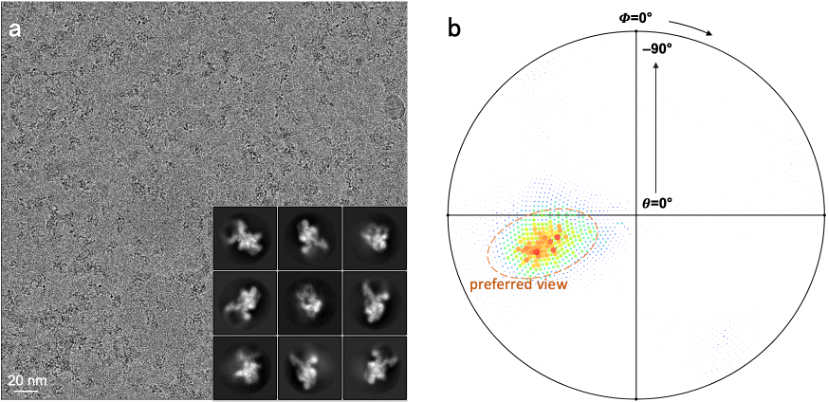


**Supplementary Fig. 12. Cryo-EM analysis of Ll.LtrB RNP on conventional graphene support.** **a**. A representative cryo-EM micrograph of Ll.LtrB RNP particles on conventional graphene. 2,090 micrographs were collected for orientational distribution analysis. The inset displayed the 2D classification results. **b**. The Euler angle distribution of Ll.LtrB RNP particles. The preferred view was labelled by dotted circle. Source data are provided as a Source Data file.

**Supplementary Table 1 Comparison of L1.LtrB RNP complex reconstruction results**

| Sample | Wildtype^16^ | ∆A mutant^17^ | Triad mutant^17^ | | Wildtype (this work) | |
| --- | --- | --- | --- | --- | --- | --- |
| Microscope | FEI Titan Krios (300 kV) | FEI Titan Krios (300 kV) | | FEI Titan Krios  (300 kV) | | FEI Titan Krios (300 kV) |
| Camera | K2 | K2 | K2 | | K3 | |
| Micrographs | 2,266 | 9,358 | 8,741 | | 1,285 from NFG;  1,472 from SFG | |
| Pixel size (Å) | 1.306 | 1.306 | 1.306 | | 0.97 | |
| Particle number | 450,296 | 231,724 | 351,541 | | 399,660 | |
| Resolution | 4.2 Å | 5.1 Å | 8.4 Å | | 3.2 Å | |
| Grids | QUANTIFOIL^®^ R 1.2/1/3 Cu-Rh 300 mesh, coated with thin-carbon film | QUANTIFOIL^®^ R 1.2/1/3 Au 300 mesh | QUANTIFOIL^®^ R 1.2/1/3 Au 300 mesh | | QUANTIFOIL^®^ R 1.2/1/3 Au 300 mesh, coated with SFG or NFG | |

**Supplementary Table 2 Cryo-EM data collection, refinement and validation statistics**

|  | Ll.LtrB RNP  (EMDB-33039; PDB-8H2H) |
| --- | --- |
| **Data collection and processing** |  |
| Magnification | 29,000 |
| Voltage (kV) | 300 |
| Electron exposure (e–/Å^2^) | 50 |
| Defocus range (μm) | 0.8-2.5 |
| Pixel size (Å) | 0.97 |
| Symmetry imposed | C1 |
| Initial particle images (no.) | 1,505,481 |
| Final particle images (no.) | 399,660 |
| Map resolution (Å)  FSC threshold | 3.2 |
| Map resolution range (Å) | 3.1-7.5 |
|  |  |
| **Refinement** |  |
| Initial model used (PDB code) | 5G2X |
| Model resolution (Å)  FSC threshold | 3.5  0.5 |
| Model resolution range (Å) | 2.7-3.5 |
| Map sharpening *B* factor (Å^2^) | -146.151 |
| Model composition  Non-hydrogen atoms  Protein residues  Ligands | 20,016  599  704 |
| *B* factors (Å^2^)  Protein  Ligand | 8.25/71.76/35.04  / |
| R.m.s. deviations  Bond lengths (Å)  Bond angles (°) | 0.025  2.618 |
| Validation  MolProbity score  Clashscore  Poor rotamers (%) | 2.79  16.74  4.04 |
| Ramachandran plot  Favored (%)  Allowed (%)  Disallowed (%) | 87.60  12.23  0.17 |
