# Supplementary material for: Functionalized graphene grids with various charges for single-particle cryo-EM
Source: Nat Commun. 2022 Nov 7;13:6718. doi: 10.1038/s41467-022-34579-w (PMC9640669; doi:10.1038/s41467-022-34579-w)
Supplement: Supplementary file 2 — Reporting Summary [file 41467_2022_34579_MOESM2_ESM.pdf]

nature research

Corresponding author(s): Hong-Wei Wang

Last updated by author(s): Sep 14, 2022

Reporting Summary

Nature Research wishes to improve the reproducibility of the work that we publish. This form provides structure for consistency and transparency in reporting. For further information on Nature Research policies, see our Editorial Policies and the Editorial Policy Checklist.

Statistics

For all statistical analyses, confirm that the following items are present in the figure legend, table legend, main text, or Methods section.

n/a	Confirmed	
☐	☒	The exact sample size (*n*) for each experimental group/condition, given as a discrete number and unit of measurement
☐	☒	A statement on whether measurements were taken from distinct samples or whether the same sample was measured repeatedly
☒	☐	The statistical test(s) used AND whether they are one- or two-sided<br>*Only common tests should be described solely by name; describe more complex techniques in the Methods section.*
☒	☐	A description of all covariates tested
☒	☐	A description of any assumptions or corrections, such as tests of normality and adjustment for multiple comparisons
☐	☒	A full description of the statistical parameters including central tendency (e.g. means) or other basic estimates (e.g. regression coefficient) AND variation (e.g. standard deviation) or associated estimates of uncertainty (e.g. confidence intervals)
☒	☐	For null hypothesis testing, the test statistic (e.g. F, t, r) with confidence intervals, effect sizes, degrees of freedom and P value noted<br>*Give P values as exact values whenever suitable.*
☒	☐	For Bayesian analysis, information on the choice of priors and Markov chain Monte Carlo settings
☒	☐	For hierarchical and complex designs, identification of the appropriate level for tests and full reporting of outcomes
☒	☐	Estimates of effect sizes (e.g. Cohen's *d*, Pearson's *r*), indicating how they were calculated

Our web collection on statistics for biologists contains articles on many of the points above.

Software and code

Policy information about availability of computer code

Data collection	We used SerialEM software (version 3.8) to collect cryo-EM datasets, described in the paper of Mastronarde (Mastronarde, D.N. 2005. Automated electron microscope tomography using robust prediction of specimen movements. J. Struct. Biol. 152:36-51).
Data analysis	We used MotionCor2 (version 1.1.0) to correct the beam-induced motion of cryo-EM micrographs and used RELION (version 3.1.3) to perform 3D reconstruction. The CTF values of these motion-corrected micrographs were determined by CTFFIND4 algorithm (version 4.15). The structural analysis was performed in UCSF Chimera (version 1.13.1). For cryo-ET reconstruction, we used IMOD (version 4.11) to align and reconstruct the tomograms. All these softwares are open-source. The Euler angle distribution of particles were analyzed using the dataset generated by RELION.

For manuscripts utilizing custom algorithms or software that are central to the research but not yet described in published literature, software must be made available to editors and reviewers. We strongly encourage code deposition in a community repository (e.g. GitHub). See the Nature Research guidelines for submitting code & software for further information.

Data

Policy information about availability of data

All manuscripts must include a data availability statement. This statement should provide the following information, where applicable:

- Accession codes, unique identifiers, or web links for publicly available datasets
- A list of figures that have associated raw data
- A description of any restrictions on data availability

Data supporting the findings in this manuscript are available from the corresponding authors upon requests. The cryo-EM density of Ll.LtrB has been deposited in the Electron Microscopy Data Bank (EMDB) under accession number EMD-33039 [https://www.ebi.ac.uk/emdb/EMD-33039], and the associated PDB code 7D1A

[https://www.rcsb.org/structure/7D1A] has been previously published. The source data underlying Figure 1c, Figure 2d-f, Figure 3a, c, d, f, g, and i and, Figure 4a and c, and Supplementary Fig. 3, Supplementary Fig. 9d-f, Supplementary Fig. 11 and Supplementary Fig. 12b are provided as a Source Data file.

Field-specific reporting

Please select the one below that is the best fit for your research. If you are not sure, read the appropriate sections before making your selection.

☒ Life sciences ☐ Behavioural & social sciences ☐ Ecological, evolutionary & environmental sciences

For a reference copy of the document with all sections, see nature.com/documents/nr-reporting-summary-flat.pdf

Life sciences study design

All studies must disclose on these points even when the disclosure is negative.

Sample size	The molecular weight of 20S proteasome is about 700kDa, ribosome is about 1.7MDa and Ll.LtrB is about 350 kDa. For cryo-EM reconstruction, we collected 1,285 micrographs for Ll.LtrB on NFG and 1,472 micrographs for Ll.LtrB on SFG, and the combined particle number used for the final reconstruction of Ll.LtrB was 399,660, and the resolution was 3.2-Å, estimated by the Fourier Shell Correction (FSC)=0.143 cutoff criteria. For Ll.LtrB RNP on graphene, we collected 2,090 micrographs and the particle number on conventional graphene was 55,783, and the resolution was ~6.0 Å. The image number used for cryo-EM analysis normally ranges from several hundred to several thousand, which is well-accepted sample size criterion in the field.<br>To characterize the cleanliness of graphene grid, we imaged 141 holes and found 123 holes (~87%) with no or no-more-than-five contamination spots, and 98 holes (~70%) with no or no-more-than-three contamination spots, where the contamination spot is defined as a stain spot with an area of > 400 square nanometers. For EM grids (Quantifoil, 300 mesh, 1.2/1.3), ~100 holes covers an area of ~625 square micron which are sufficient for cleanliness characterization of graphene grids because the micrographs we took when collecting cryo-EM dataset is only about 0.2 square micron.
Data exclusions	For cryo-EM reconstruction, particles grouped in bad classes with poorly defined features were excluded, because these particles were normally denatured or dissociated samples, which were harmful for high-resolution 3D reconstruction.
Replication	To test the general application of the graphene membrane, we used three different testing samples: 20S proteasome, ribosome, and Ll.LtrB RNP. To calculate the water contact angles, we performed more than 3 times measurements (as demonstrated in the figure legend).
Randomization	Samples were allocated randomly in the structural determination in Relion (version 3.1.3).
Blinding	For cryo-EM reconstruction in Relion (version 3.1.3), particles were randomly divided into two parts, and used for 3D structure determination. The consistence of structures generated by these two sub-datasets was used for the blinding test.

Reporting for specific materials, systems and methods

We require information from authors about some types of materials, experimental systems and methods used in many studies. Here, indicate whether each material, system or method listed is relevant to your study. If you are not sure if a list item applies to your research, read the appropriate section before selecting a response.

Materials & experimental systems

n/a	Involved in the study
☒	☐ Antibodies
☒	☐ Eukaryotic cell lines
☒	☐ Palaeontology and archaeology
☒	☐ Animals and other organisms
☒	☐ Human research participants
☒	☐ Clinical data
☒	☐ Dual use research of concern

Methods

n/a	Involved in the study
☒	☐ ChIP-seq
☒	☐ Flow cytometry
☒	☐ MRI-based neuroimaging
