# Supplementary figures and images for: Functionalized graphene grids with various charges for single-particle cryo-EM
Source: Nat Commun. 2022 Nov 7;13:6718. doi: 10.1038/s41467-022-34579-w (PMC9640669; doi:10.1038/s41467-022-34579-w)

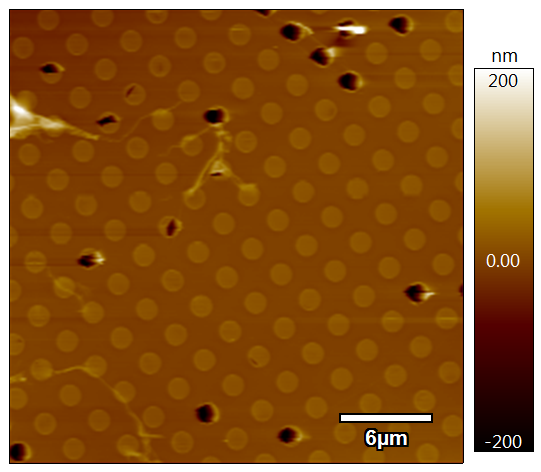

Supplement: Supplementary file 3 — Source Data [file 41467_2022_34579_MOESM3_ESM.zip › Source Data/supplementaryFig3.TIF]
